# Can Virtual Reality Simulation Trainers Provide Transferable Skills in Knee Arthroscopy?

**DOI:** 10.7759/cureus.99297

**Published:** 2025-12-15

**Authors:** Jefferson George, Indhu Poomalai, Surya Malasani, Vivek Thakker, Karl J New

**Affiliations:** 1 Trauma and Orthopaedic Surgery, University Hospitals of Leicester NHS Trust, Leicester, GBR; 2 Clinical Sciences, University of South Wales, Wales, GBR

**Keywords:** knee arthroscopy, knee surgery sports traumatology and arthroscopy, simulation training, virtual, virtual reality

## Abstract

Virtual reality (VR) simulation is emerging as a high-fidelity training tool for knee arthroscopy, providing a risk-free learning environment. This study systematically evaluates current evidence on the effectiveness of VR simulation training in providing transferable skills for knee arthroscopy and the effects on operative time, complication rates and clinical outcomes. A systematic review of nine databases identified studies published until September 2024 using search terms “knee arthroscopy” and “simulation” or “simulator.” Key outcomes included time to task completion, operative time, Arthroscopic Surgical Skill Evaluation Tool (ASSET) scores, path length and validity measures. Previous reviews were assessed using the A Measurement Tool to Assess Systematic Reviews 2 (AMSTAR-2) tool. Risk of bias was evaluated with ROB-2 and ROBINS-I tools. A random-effects meta-analysis was performed to account for study heterogeneity. Nine studies (seven randomised controlled trials (RCTs) and two observational studies) involving 327 participants across eight countries were included. Seven studies confirmed skill transfer to cadaveric models; only one showed intraoperative (in vivo) transfer.

Compared with controls, the VR-trained participants showed significant improvement in time to completion (mean difference (MD): -87.58 seconds; p=0.010; 95% confidence interval (CI): -153.88 to -21.28 seconds). No difference was seen compared to other methods of simulation training in time (MD: 2.97 seconds; p=0.90; 95% CI: -44.35 to 50.29) and ASSET scores (MD: 0.76; p=0.42; 95% CI: -1.09 to 2.61). Across the seven RCTs, the VR-trained groups showed shorter procedure times, improved movement economy and higher global scores compared with the non-VR-trained groups.

VR simulation is a potentially effective tool for knee arthroscopic training, providing a safe learning environment, but there remains scope to improve and validate its effectiveness in vivo. While evidence supports skill transfer to simulated cadaveric and simulated environments, further research is needed to validate in vivo transfer, assess clinical impact and evaluate cost-effectiveness and scalability for widespread curriculum integration.

## Introduction and background

Knee arthroscopy is one of the most common orthopaedic procedures performed worldwide for diagnostic and therapeutic interventions of intra-articular knee pathologies [1]. However, this minimally invasive technique poses a unique learning challenge due to its reliance on visuospatial coordination and psychomotor skills [2]. Traditional models of training, increasingly constrained by evolving work-hour regulations, ethical concerns and costs [3,4], may limit and impede arthroscopic skill acquisition in early trainees. To mitigate this, the last two decades have seen a growing trend towards surgical training outside the operating theatres and the incorporation of simulation training models in orthopaedic training. Virtual reality (VR) simulation has already been integrated into training pathways in several surgical specialties, and in orthopaedics, it has the potential to prepare trainees prior to operating theatres and cadaveric workshops by allowing them to develop foundational skills in a risk-free environment.

Simulation training in arthroscopy is gradually emerging as a promising alternative to develop technical competence by offering an immersive and safe environment for repetitive practice [5]. Broadly, there are three types of simulators: benchtop (BT) trainers, animal or cadaveric simulation models and virtual reality (VR) simulators [6]. Of the three, VR simulators are fast becoming the adjunct to arthroscopic training by nature of their high fidelity, instant objective feedback and little or no reliance on supervision. VR trainers objectively record several performance metrics, such as completion time, path length, error rates and economy of movement by tracking the user’s interaction with the simulated environment. This places VR simulators as a scalable and data-driven solution in surgical curricula across training programmes [7,8].

The challenge to the adoption of a learning technology is its validity or its ability to accurately teach the phenomenon it is intended to teach [9]. Validity ensures a standardised design for the delivery and evaluation of the intervention, through evidence-based practices [10]. Validity also controls internal and external factors such as confounding and generalisability, so that an intervention may be impactful in a real-world setting. In the context of arthroscopic simulation trainers, most studies focus on face, content and construct validities to assess the learning potential of the trainer. However, the ultimate test of a simulator trainer’s validity would be its ability to provide transferable skills to the intended setting, such as the operating theatres, also known as transfer validity [11].

Systematic reviews have previously been conducted to investigate the role of simulators in arthroscopic simulation training [11-14]. However, all these reviews have suffered from certain drawbacks and methodological shortcomings. Firstly, the most recent review only outlined studies published up to 2019 [14]. The inherent evolving nature of this technology-driven educational tool has led to frequent research in this field. Moreover, previous reviews have not exclusively investigated the validity of virtual reality simulation trainers with a focus on knee arthroscopy. Most reviews included all forms of simulation, were not specific to the knee joint and were not specific to virtual reality training. The focus of this review was to collate the most up-to-date evidence on the transfer validity of VR simulation training in knee arthroscopy.

This review specifically seeks to determine whether VR simulation training demonstrates evidence of transfer validity to cadaveric, simulated or clinical settings in knee arthroscopy.

## Review

Materials and methods

Search Strategy

**Figure 1:**
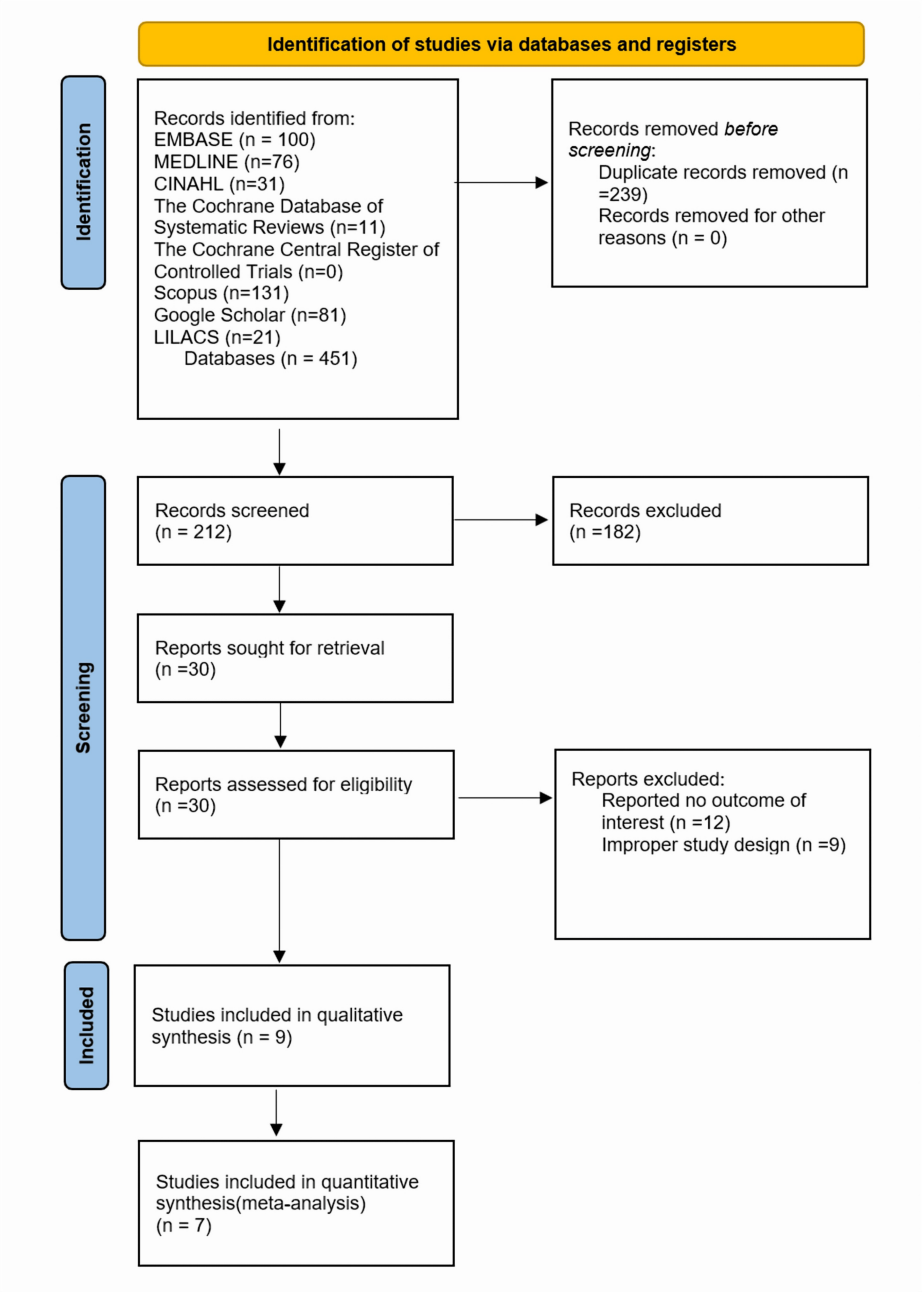
PRISMA flowchart illustrating the systematic review and meta-analysis process The chart provides a visual representation of the study selection process, including the identification, screening, eligibility assessment and inclusion of articles for analysis. EMBASE: Excerpta Medica Database, MEDLINE: Medical Literature Analysis and Retrieval System Online, CINAHL: Cumulative Index to Nursing and Allied Health Literature, CENTRAL: Cochrane Central Register of Controlled Trials, LILACS: Latin American and Caribbean Health Sciences Literature, PRISMA: Preferred Reporting Items for Systematic Reviews and Meta-Analyses

This systematic review was conducted following a protocol registered a priori on the Prospective Register of Systematic Reviews (PROSPERO) (CRD420250569172) [15]. The review was in strict adherence to the guidelines by the Preferred Reporting Items for Systematic Reviews and Meta-Analyses (PRISMA) statement [16]. Our search spanned Medical Literature Analysis and Retrieval System Online (MEDLINE), Excerpta Medica Database (EMBASE), Cochrane, Cochrane Central Register of Controlled Trials (CENTRAL), Cumulative Index to Nursing and Allied Health Literature (CINAHL), Google Scholar, Scopus, Web of Science and Latin American and Caribbean Health Sciences Literature (LILACS) databases for studies unrestricted by publication date. We used text words and MeSH terms, combining key terms such as “knee arthroscopy,” “simulation training,” “virtual reality” and various terms related to arthroscopic surgery, high-fidelity and training covering literature up to October 2024. No date limits were applied to the search strategy; however, given the recent emergence of VR simulation technology, all eligible studies were published within the last 15 years. The PRISMA flowchart is summarised in Figure 1.

Type of Studies Included

Studies assessing the validity of VR simulation trainers for knee arthroscopy with relevant outcomes for transfer validity encompassing randomised controlled trials (RCTs), non-randomised controlled trials and prospective cohort studies were included. Studies not involving virtual reality simulation trainers and focusing on other types of arthroscopy or medical procedures will be excluded. Reviews, commentaries and editorials without original data were also outside the scope of this review. Studies with inadequate information on study design, sample size or data analysis were excluded. Duplicates were eliminated.

Population (P): The population included all participants learning knee arthroscopy (including medical students, junior doctors, orthopaedic residents or individuals with limited prior arthroscopy experience).

Intervention (I): The intervention was training using a virtual reality (VR) knee arthroscopy simulator.

Comparator (C): Comparators included any comparator defined within the included primary studies, including no VR training/standard curriculum, benchtop or box trainers or cadaveric training.

Outcomes (O): Outcomes included measures of transfer validity.

Selection of Studies

The studies from the electronic database searches were screened and selected using Rayyan software [17]. Two authors independently reviewed titles and abstracts to identify eligible studies. They independently evaluated the full texts of studies meeting the inclusion criteria to confirm eligibility. Disagreements were resolved by the senior author. This screening process, including exclusion reasons, was meticulously documented using a PRISMA flow diagram.

Data Extraction and Management

Data extraction was performed independently by two reviewers using a standardised form and cross-verified for accuracy. Any discrepancies or missing data were resolved through consensus or by contacting study authors when necessary. Collected data included date of publication, journal, country, participant demographics (level of expertise, age, gender and previous experience in VR), intervention, simulator model, methodological characteristics of each study and outcomes such as time to task completion, Arthroscopic Surgical Skill Evaluation Tool (ASSET) score, path length and any other measures assessed at baseline and follow-up. Data analysis aspects (statistical methods, effect size, confidence intervals, heterogeneity and publication bias) and the study’s strengths and limitations were also recorded. Control conditions were defined by the individual studies and typically included no VR training, standard educational activities or alternative simulation modalities such as benchtop or cadaveric training. These comparator groups were extracted as reported to preserve the methodological integrity of each study and ensure accurate synthesis.

Risk of Bias and Quality Assessment

**Figure 2:**
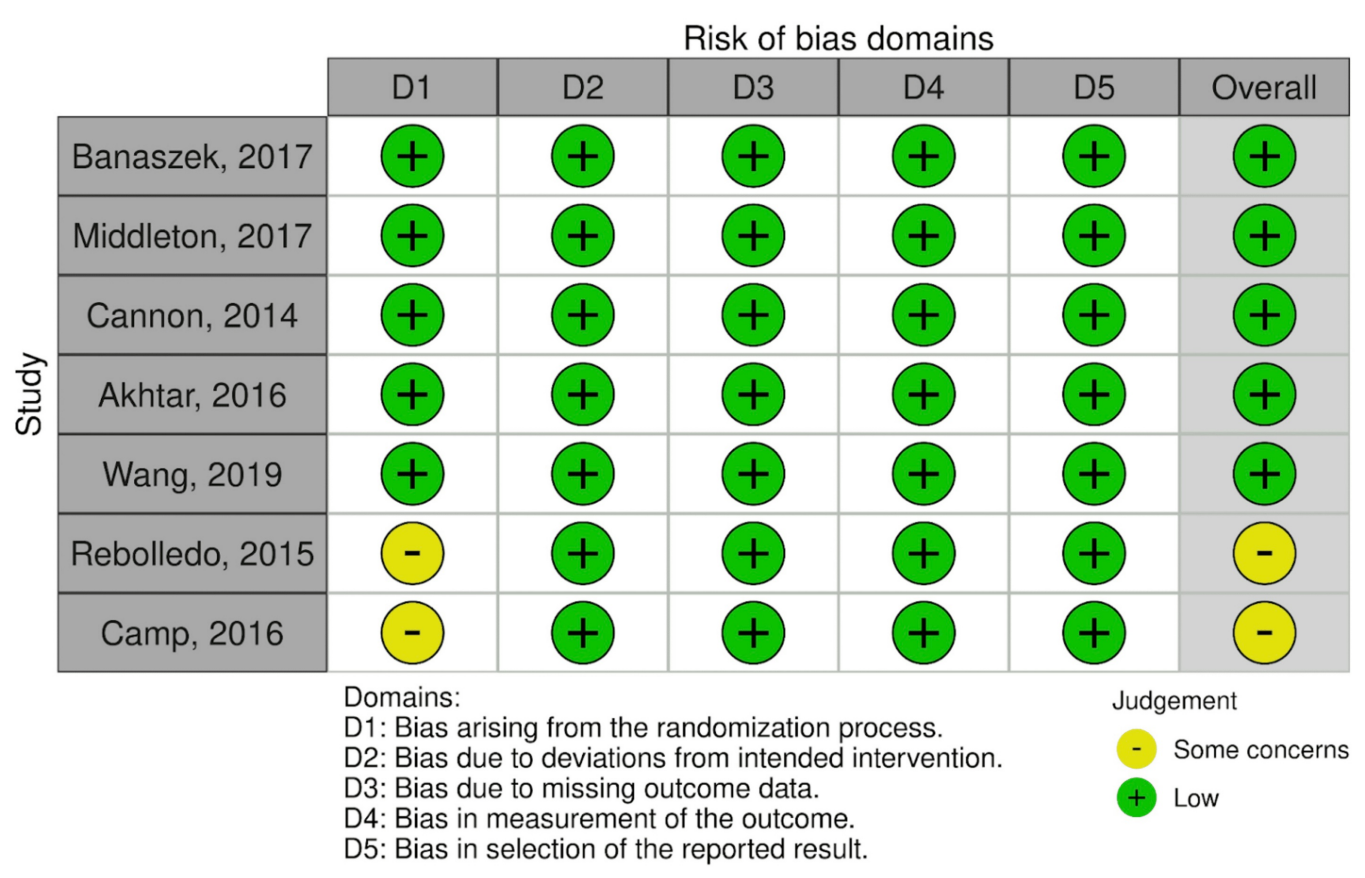
Risk of bias assessment of included RCTs using the ROB-2 tool Included studies: Banaszek et al. (2017) [19], Middleton et al. (2017) [20], Cannon et al. (2014) [21], Akhtar et al. (2016) [22], Wang et al. (2019) [23], Rebolledo et al. (2015) [24] and Camp et al. (2016) [25] Risk of bias assessment for the seven included RCTs across the five ROB-2 domains: D1: bias arising from the randomisation process, D2: bias due to deviations from intended interventions, D3: bias due to missing outcome data, D4: bias in measurement of the outcome, and D5: bias in selection of the reported result. Each circle indicates the reviewer’s judgement: green (“low risk”) and yellow (“some concerns”). RCT: randomised controlled trial

**Figure 3:**
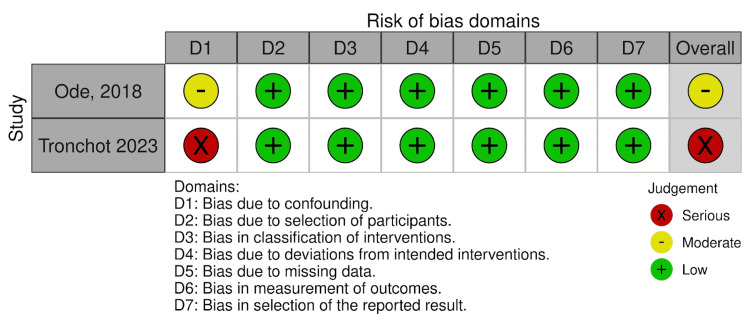
Risk of bias assessment of non-randomised studies using ROBINS-I tool Included studies: Ode et al. (2018) [26] and Tronchot et al. (2023) [27] Risk of bias assessment for the two observational studies included in this review, evaluated using the ROBINS-I tool across seven domains: D1: bias due to confounding, D2: bias due to selection of participants, D3: bias in classification of interventions, D4: bias due to deviations from intended interventions, D5: bias due to missing data, D6: bias in measurement of outcomes, and D7: bias in the selection of the reported result. Judgement categories are represented as green (“low risk”), yellow (“moderate risk”) and red (“serious risk”).

Previous reviews on this topic were identified and their flaws noted. The two most recent reviews were critically appraised using A Measurement Tool to Assess Systematic Reviews 2 (AMSTAR-2) [18] to further justify a new review on the topic (Table 1). Risk of bias was assessed using Cochrane’s ROB 2.0 for RCTs and Cochrane’s ROBINS-I for non-randomised studies by two independent authors, with discrepancies resolved through arbitration by a senior reviewer (Figure 2 and Figure 3).

**Table 1:** Assessment of previous systematic reviews using the AMSTAR-2 tool AMSTAR-2: A Measurement Tool to Assess Systematic Reviews 2

Author and year	Critical flaws	Non-critical weaknesses	Overall confidence rating
Capitani et al. (2021) [14]	5	2	Critically low
Polce et al. (2020) [12]	3	2	Critically low

Data Analysis

All statistical analyses were performed using RevMan version 5.4 (The Cochrane Collaboration, London, UK). We extracted the effect sizes of outcomes recorded such as time to completion, ASSET scores and any other metrics recorded from the included studies. A random-effects model was utilised to account for heterogeneity arising from different simulators used and outcomes recorded and to pool the mean difference along with 95% confidence intervals (CIs). A random-effects model was selected due to anticipated heterogeneity in study design and outcome measurement, in line with Cochrane recommendations. Forest plots were generated to assess the pooled results. Heterogeneity was evaluated using the Higgins I-squared test in accordance with the Cochrane Handbook.

Results

A total of nine studies were found eligible for this review, of which seven were randomised controlled studies and two were observational studies, including 327 participants in total. Two studies were multicentre studies, while seven studies were single-centre investigations. While only one study focused on in vivo transfer validity, all other studies focused on transfer validity to cadaveric knees, cadaveric wrists or between simulators [19-25]. The characteristics of the included studies are summarised in Table 2.

**Table 2:** Characteristics of included studies evaluating VR simulation in knee arthroscopy AANA: Arthroscopy Association of North America, AAOS: American Academy of Orthopaedic Surgeons, ABOS: American Board of Orthopaedic Surgery, BT: benchtop, PGY: postgraduate year, RCT: randomised controlled trial, VR: virtual reality

Study	Year	Name of VR simulator	Population	Prior experience in arthroscopy	Comparison	Control group	Transfer to a live patient	Level of evidence	Single centre/multicentre	Funding source (private/public/not reported)	Country
Banaszek et al. [19]	2017	Arthro VR (GMV)	40 medical students in 3 groups: VR group (n=16), BT group (n=16) and control group (n=8)	No	VR versus BT	Yes	No (on cadaveric knee)	I (RCT)	Single	Institutional (public)	Canada
Middleton et al. [20]	2017	ArthroS VR Simulator (VirtaMed AG, Zurich, Switzerland)	17 medical students and interns (8 in the VR group and 9 in the BT group)	No	VR versus BT	No control	No	I (RCT)	Single	Partly funded by McLaren Applied Technologies	UK
Cannon et al. [21]	2014	ArthroSim VR Knee Simulator (Touch of Life Technologies, Aurora, Colorado)	48 PGY-3 orthopaedic residents (27 in the VR group and 21 in the control group)	Varied prior arthroscopic experience (range: 1-100 cases, mean: 28 cases per resident)	VR versus traditional training	Yes	Yes	I (RCT)	Multicentre (7 institutions)	Public (funded by the AAOS, AANA and ABOS)	USA
Akhtar et al. [22]	2016	LapMentor VR Simulator (for laparoscopy) and Arthro Mentor VR simulator (for arthroscopy) (Simbionix, Cleveland, OH, USA)	72 medical students in 4 groups: 2 control groups (n=16 each) and 2 training groups (n=20 each)	No	VR versus no training	Yes	No	I (RCT)	Single	Not mentioned	UK
Wang et al. [23]	2019	ArthroVision Virtual Reality Simulator (Swemac, Linköping, Sweden)	28 medical and premedical students (n=14 in each group; training and control)	No	VR vs no training	Yes	No (on cadaveric knee)	I (RCT)	Single	Public (ABOS education grant)	USA
Rebolledo et al. [24]	2015	Insight Arthro VR Simulator (GMV)	14 PGY-1/PGY-2 orthopaedic surgery residents: VR simulator group (n=8) and didactic group (n=6)	No	VR versus no training	Yes, two hours of didactic lectures	No (on cadaveric knee)	I (RCT)	Single	Public	USA
Camp et al. [25]	2016	ArthroSim Virtual Reality Arthroscopy Simulator (Touch of Life Technologies)	45 orthopaedic residents (PGY-1 to PGY-5); 3 groups: VR simulator, cadaver and control (n=15 per group)	No	VR versus cadaveric training	Yes	No (cadaveric knee)	I (RCT)	Single	Public	USA
Ode et al. [26]	2018	ArthroSim Knee Arthroscopy Simulator (Touch of Life Technologies)	N=27: 10 PGY-0/1, 10 juniors PGY-2/3 and 7 seniors PGY-4/5	Yes, 28 knee arthroscopies per participant (range: 1-100)	VR training only	No control	No (cadaveric wrist)	II	Single	Public (funded by the Winkler Orthopaedic Fellowship Fund)	USA
Tronchot et al. [27]	2023	VirtaMed ArthroS™ Hybrid VR Simulator (Schlieren)	N=36 (18 in each group: VR and non-VR) (orthopaedic residents PGY-1 and PGY-2)	Zero or minimal experience	VR training versus no training	Yes, traditional training, including lectures, anatomy laboratories and supervised practice using benchtop arthroscopy models	No (cadaveric knee)	II	Multicentre (5 institutions)	Public	France

Several different outcome measures were reported by each study, with the most frequent being time to completion [19-26] and the Arthroscopic Surgical Skill Evaluation Tool (ASSET) score [23-27]. There was heterogeneity in the outcome measures recorded, including different types of global rating scales (GRS), motion analysis metrics and simulator-specific metrics such as path length, probe roughness, individualised procedural checklists, number of attending surgeon interventions and Injury Grading Index (Table 3). Most of the outcome measures reported across the included studies represented surrogate indicators of technical performance rather than true clinical endpoints.

**Table 3:** Summary of types of VR simulators used, transfer validities tested and outcomes/metrics recorded ASSET: Arthroscopic Surgical Skill Evaluation Tool, IGI: Injury Grading Index, VR: virtual reality

Simulator	Number of studies
ArthroS VR Simulator (VirtaMed AG, Zurich, Switzerland)	2
ArthroSim VR Knee Simulator (Touch of Life Technologies, Aurora, Colorado)	3
ArthroVision Virtual Reality Simulator (Swemac, Linköping, Sweden)	1
Arthro VR Simulator (GMV)	2
ArthroMentor VR Simulator (Simbionix, Cleveland, OH, USA)	1
Transfer validity	
Transfer of skills to cadaver	6
Transfer of skills to live patient	1
Transfer of skills between simulators	2
Outcomes recorded	
ASSET score	4
Time to completion	6
Global rating scale	3
Simulator metrics (e.g., path length, probe/camera roughness and speed)	3
Procedural checklist score (visualisation and probing accuracy)	1
Motion analysis (hand movements and sensor-based kinematics)	2
Subjective assessment score	2
Number of interventions by attending surgeons	1
IGI	1

The outcomes and results are summarised in Table 4.

**Table 4:** Summary of outcomes, validity and transfer measures in the included studies ASSET: Arthroscopic Surgical Skill Evaluation Tool, BT: benchtop, GRS: global rating scale, IGI: injury grading index, VR: virtual reality

Study	Year	Name of VR simulator	Measured outcomes	Method	Results	Conclusion
Banaszek et al. [19]	2017	Arthro VR (GMV)	GRS, the 14-point arthroscopic checklist time to task completion, secondary outcomes: motion analysis (VR-specific metrics): camera distance and roughness, and probe distance and roughness	Diagnostic arthroscopy first in both VR and BT simulators (cross-over design), followed by independent practice in randomly assigned simulator over five weeks; post-training tests included diagnostic arthroscopy on both simulators, diagnostic knee arthroscopy and partial medial meniscectomy on cadaveric specimens.	Post-training GRS scores were 18.7 (VR), 15.6 (BT) and 9.3 (control), indicating a significant improvement in the VR group (p<0.001). On the 14-point arthroscopic checklist, post-training scores were 13.6 (VR), 11.0 (BT) and 8.0 (control), with the VR group achieving the largest mean improvement of +6.2 points (p<0.001). Procedural efficiency improved markedly in both trained groups; for cadaveric tasks, VR-trained participants completed tasks in 95 seconds compared to 73 seconds (BT) and 182 seconds (control), with both trained groups outperforming controls (p<0.001). Additionally, VR participants exhibited better motion control metrics, such as reduced camera distance (1,375 mm post-training versus 3,475 mm pre-training; p=0.033). For the surprise skill transfer task (partial meniscectomy), 31% of VR participants successfully completed the task within four minutes, compared to 0% for BT and control groups (p=0.014).	Both VR and BT simulations were effective, but VR simulation provided superior training outcomes, especially in skill transfer to realistic scenarios.
Middleton et al. [20]	2017	ArthroS VR Simulator (VirtaMed AG, Zurich, Switzerland)	Motion analysis: total time and number of hand movements (assessed using wireless sensors), GRS	Baseline diagnostic arthroscopy task on both simulators, followed by three training sessions on the allocated simulator over 2-3 weeks; post-training assessment on both simulators	In the VR group, the total time decreased from 229.5 seconds (pre-training) to 127.3 seconds (post-training) (p=0.012), hand movements reduced from 228.0 to 141.5 (p=0.012) and GRS scores improved from 8.5 to 18.5 (p=0.012). In the BT group, the total time decreased from 244.0 seconds (pre-training) to 65.2 seconds (post-training) (p=0.008), hand movements reduced from 264.0 to 87.0 (p=0.008) and GRS scores improved from 12.5 to 25.0 (p=0.012). On final comparison, on the BT simulator, BT-trained participants significantly outperformed VR-trained participants (total time: 65.2 seconds (BT) versus 134.4 seconds (VR) (p=0.002), hand movements: 87.0 (BT) versus 158.5 (VR) (p=0.008) and GRS: 25.0 (BT) versus 18.0 (VR) (p=0.002)). On the VR simulator, no significant differences were observed.	Both simulators significantly improved arthroscopic skills (p<0.05). BT-trained participants showed significantly better final performance on the BT simulator compared to VR-trained participants (p<0.05). BT training skills transferred effectively to the VR simulator, but VR-trained skills did not transfer as effectively to the BT simulator.
Cannon et al. [21]	2014	ArthroSim VR Knee Simulator (Touch of Life Technologies, Aurora, Colorado)	Procedural checklist score (with visualisation and probing scales), GRS, duration of final task and number of interventions by attending surgeons	The VR group practiced diagnostic knee arthroscopy on a simulator to achieve a predefined proficiency level, while the control group continued traditional training; post-training assessment was diagnostic knee arthroscopy in vivo with assessment by a blinded rater	The simulator-trained group demonstrated significant improvements in procedural checklist scores (63% versus 52%; p=0.031), particularly in probing tasks (64% versus 48%; p=0.016). No significant differences in visualisation scores (61% versus 58%, p=0.34) or global rating scores (p=0.061 with outlier, p=0.04 without). Time to complete the procedure was not significantly different (972 versus 929 seconds; p=0.673). Simulator-trained residents required fewer corrective interventions (average: 2.5 versus 4 per resident, p=0.044).	The VR simulation group showed better skill acquisition, particularly in probing tasks. No significant difference was noted in other outcomes.
Akhtar et al. [22]	2016	LapMentor VR Simulator (for laparoscopy) and ArthroMentor VR Simulator (for arthroscopy) (Simbionix, Cleveland, OH, USA)	Total time to complete tasks, instrument path length (left and right hand), instrument speed (left and right hand) and face validity	Training groups performed a task, underwent training on the alternate simulator (VR arthroscopy and VR laparoscopy) and retested on the initial task; control groups performed task without training	The laparoscopy-trained group (group 4) showed the greatest improvement in task completion time, reducing it by 48% on the arthroscopy simulator (541 seconds to 281 seconds; p<0.05). The arthroscopy-trained group (group 3) reduced task time by 24.4% (229 seconds to 173 seconds). Training groups also exhibited better economy of movement, with significant reductions in left-hand path length (group 4: 36.1%, group 3: 17.6%) and notable increases in left- and right-hand instrument speed (group 4: +34.8% and +59.3%, respectively). Both simulators were rated highly for realism, tactile feedback and utility.	Generic minimally invasive surgical skills learned on a VR laparoscopy simulator are transferable to VR arthroscopy simulators.
Wang et al. [23]	2019	ArthroVision Virtual Reality Simulator (Swemac, Linköping, Sweden)	ASSET, task completion time and simulator metrics such as path length and task-specific time measures	The training group completed multiple sessions on the VR simulator until a predefined proficiency threshold; both groups performed a pre-test and post-test on the simulator, followed by a diagnostic arthroscopy on the cadaveric knee and shoulder	Significant difference in simulator task completion time was seen in the simulator-trained group compared to the control group (p<0.05) during the post-test. The mean ASSET scores showed no difference for cadaveric knee arthroscopy: 19.25±2.46 for the simulator-trained group and 18.00±7.43 for controls (p=0.55).	Simulator training significantly improved simulator task performance but did not transfer to cadaveric diagnostic arthroscopy skills.
Rebolledo et al. [24]	2015	Insight Arthro VR Simulator (GMV)	Time to completion, IGI, diagnostic arthroscopy checklist and improvement in performance trends	2 hours of training on the VR simulator; both groups test on the cadaveric knee	The simulator-trained group completed the knee arthroscopy tasks faster, with a mean time of 5.1±1.8 minutes compared to 8.0±4.1 minutes for the didactic group (p=0.09). Similarly, the Injury Grading Index (IGI) scores, which assess instrument handling and intra-articular injury, were lower for the simulator group (4.0 ± 1.0) than the didactic group (5.3 ± 1.5, p = 0.08).	VR simulator training showed a trend towards better performance in knee arthroscopy tasks compared to didactic training, although the differences were not statistically significant.
Camp et al. [25]	2016	ArthroSim Virtual Reality Arthroscopy Simulator (Touch of Life Technologies)	ASSET score test completion time	4 hours of practice on the simulator or cadaver; pre-test and post-test on the cadaveric knee	ASSET scores in the cadaver group increased significantly from 22.9±3.9 to 27.2±2.9, an improvement of +4.27 points (p=0.002), with a reduction in task completion time from 7:39±2:53 minutes to 4:38±1:42 minutes (p=0.002). The simulator group showed a smaller, non-significant improvement in ASSET scores from 22.5±3.1 to 24.4±3.1 (+1.92 points; p=0.096) and a modest, also non-significant, reduction in task time from 9:12±2:00 minutes to 8:44±4:23 minutes (p=0.708). The control group showed no significant change, with ASSET scores decreasing slightly from 24.4±4.6 to 24.0±2.8 (p=0.776), and task completion time barely changing from 8:04±1:20 minutes to 7:57±3:28 minutes (p=0.902). The cadaver group demonstrated the highest training efficiency, gaining 1.1 ASSET points per hour, compared to 0.5 points per hour in the simulator group.	Cadaveric training was the most effective method for improving technical skills and efficiency in diagnostic arthroscopy.
Ode et al. [26]	2018	ArthroSim Knee Arthroscopy Simulator (Touch of Life Technologies)	ASSET score, task completion percentage and time and subjective assessment of performance	VR knee arthroscopy simulation training up to predefined performance threshold; pre- and post-cadaveric wrist arthroscopy assessment	The mean ASSET scores decreased slightly from 24.7±9.7 pre-intervention to 23.5±7.0 post-intervention (p=0.36). Interns showed a modest increase in ASSET scores (+1.8 points), while junior and senior residents experienced decreases (-1.6 and -5.0 points, respectively). Task completion percentage for wrist arthroscopy tasks remained unchanged across the cohort, with no significant differences between pre- and post-intervention performance (p>0.05)	No significant improvement in total ASSET score. Limited skill transfer from VR knee simulation training to wrist arthroscopy was seen, with favourable subjective feedback from participants.
Tronchot et al. [27]	2023	VirtaMed ArthroS™ Hybrid VR Simulator (Schlieren)	ASSET score and global performance scores	Structured training on VR simulator over three weeks; pre- and post-testing on BT meniscectomy, diagnostic knee arthroscopy on cadavers and partial meniscectomy on cadavers	The VR group achieved higher global ASSET scores than the non-VR group (28.74±3.75 versus 26.37±5.55; p=0.002). For specific tasks, the VR group outperformed the non-VR group in diagnostic knee arthroscopy on cadavers (28.38±3.12 versus 26.08±5.12; p=0.048) and cadaveric partial meniscectomy (29.81±3.18 versus 26.70±5.70; p=0.01). BT meniscectomy scores also favoured the VR group (28.03±3.16 versus 26.28±4.33, p=0.077), although this was not statistically significant.	Transfer validity to BT and cadaveric knee was demonstrated.

Transfer of Skills to the Operating Theatres

One study [21] compared VR simulation training against traditional training in performing diagnostic knee arthroscopy in the operating theatres. The simulator-trained group showed superior performance in procedural checklist scores and probing skills; however, no differences were noted in all other parameters recorded, including time to completion.

Transfer of Skills to Cadaveric Arthroscopy

Two studies demonstrated strong transfer validity for VR-trained groups in cadaveric knee arthroscopy when compared with BT-trained and control groups [19] or control groups alone [27], with superior performance in outcomes such as ASSET scores, GRS scores and task checklists. However, another study [25] reported that cadaveric-trained subjects improved twice as fast compared to VR training with a greater ASSET score improvement, although VR-trained subjects still showed measurable benefit. In contrast, two other studies [23,24] failed to demonstrate a difference in performance between VR-trained and control groups in cadaveric diagnostic knee arthroscopy, suggesting a ceiling effect in simulator training and variability in transfer effectiveness. One study investigated transfer of skills to cadaveric wrist arthroscopy, where objective metrics such as ASSET scores, task completion percentage or time showed no statistically significant gains [26].

Transfer of Skills Between Simulators

Transfer of skills between benchtop (BT) trainers and VR simulation trainers was evaluated by one study [20], which showed that the BT-trained group demonstrated effective skill transfer to the alternate (VR) simulator; however, VR-trained subjects showed no significant improvement when reassessed on the benchtop model, suggesting superior psychomotor skill acquisition with benchtop simulation. Another study [22] demonstrated positive transferability in skills between a VR arthroscopic simulator and a VR laparoscopic simulator, with significantly better performance metrics in trained groups versus controls.

Time to Task Completion

**Figure 4:**
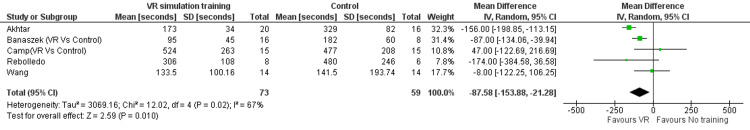
Forest plot showing mean difference in task completion time between VR-trained and control groups Included studies: Banaszek et al. (2017) [19], Akhtar et al. (2016) [22], Wang et al. (2019) [23], Rebolledo et al. (2015) [24] and Camp et al. (2016) [25] X-axis: Mean difference in time to task completion (seconds), where negative values indicate faster performance and favour VR training Y-axis: Individual studies included in the meta-analysis VR: virtual reality

Time to task completion was a metric analysed by most transfer validity studies when assessing and comparing final skills gained through VR training with other methods of training. The final task was different in each study, but always followed a period of VR-training or no training or training on a different platform, such as a benchtop simulator or cadaveric training. Data from five RCTs [19,22-25] involving 73 participants in the VR-trained group and 59 participants in the control group were analysed. Two studies [20,23] that reported their data in median and interquartile range were converted into mean and standard deviation, respectively [28,29]. A significant reduction in total time taken after VR training was noted (mean difference: -87.58; p=0.010; 95% CI: -153.88 to -21.28 seconds; I2=67%), despite heterogeneity among the studies (Figure 4).

**Figure 5:**
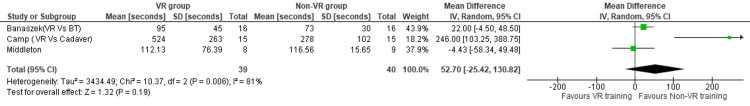
Forest plot showing mean difference in task completion time between VR-trained and non-VR groups Included studies: Banaszek et al. (2017) [19], Middleton et al. (2017) [20] and Camp et al. (2016) [25] X-axis: Mean difference in time to task completion (seconds), where negative values indicate faster performance and favour VR training Y-axis: Individual studies included in the meta-analysis VR: virtual reality

Data from three RCTs [19,20,25] involving 31 subjects in the VR-trained group and 40 subjects in the BT or cadaveric-trained group were analysed. No statistically significant difference was seen between VR training and alternate methods of training (mean difference: 52.70; p=0.19; 95% CI: -25.42 to 130.82, I2=81%) (Figure 5).

ASSET Score

**Figure 6:**
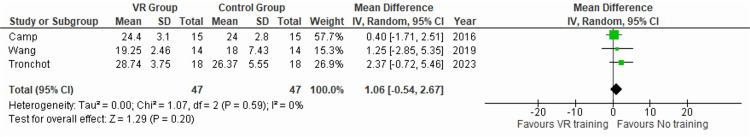
Forest plot of mean difference in ASSET scores between VR and control groups Included studies: Wang et al. (2019) [23], Camp et al. (2016) [25] and Tronchot et al. (2023) [27] X-axis: Mean difference in ASSET score (positive values favour VR training) Y-axis: Included studies ASSET: Arthroscopic Surgical Skill Evaluation Tool, VR: virtual reality

No statistically significant difference was seen between VR training groups and control groups in ASSET scores among comparable studies (mean difference: 1.06; p=0.20; 95% CI: -0.54 to 2.67; I2=0%). A sensitivity analysis was conducted by excluding one study [27] whose design had a non-randomised design. A pooled mean difference of 0.58 (95% CI: -1.30 to 2.46; p=0.55) was seen. These findings indicate that the inclusion of non-randomised data had minimal impact on the overall results (Figure 6).

Risk of Bias and Quality Assessment

The appraisal of the two most recent review papers using the AMSTAR-2 tool showed several flaws in these studies [12,14]. An overall critically low confidence rating was seen with deficiencies in items 2, 3, 6, 7 and 9. Furthermore, the most recent review was in 2021, indicating that the latest evidence up to 2024 was not considered. Using the ROB-2 tool for the seven RCTs, two studies raised some concerns, while the remaining five showed low risk of bias. Of the two observational studies, the ROBINS-I tool raised some concerns for one study [26] and exhibited a high risk of bias for the second study [27].

Discussion

This systematic review and meta-analysis supports the transfer validity of VR simulation training in knee arthroscopy. Our findings suggest that VR simulation training has the potential to provide transferable skills to surgical trainees learning knee arthroscopy, although the practical significance of these improvements remains to be confirmed in clinical settings. Several studies demonstrated improved performance in real or simulated clinical settings among VR-trained participants compared to controls. Across the included studies, two outcome measures were common and sufficiently consistent to enable comparative analysis: time to final task completion and the Arthroscopic Surgical Skill Evaluation Tool (ASSET) score. Although outcomes in simulation-based research can be divided into surrogate and clinical categories, the studies included in this review overwhelmingly reported surrogate technical metrics, with only a single study examining in vivo clinical performance. Because of this pronounced imbalance, the most comparable outcomes have been evaluated in this review.

Time to task completion, defined as the duration required to complete the final task (such as a diagnostic knee arthroscopy on a cadaver or a live patient) following a period of simulator training, showed statistically significant reductions in five studies [19,22-25] when compared with controls. However, when VR training was compared to benchtop or cadaveric training [19,20,25], a difference was not observed. This indicated comparable effectiveness between VR training and other simulation modalities. Previous reviews [12,30] that looked at the efficacy of all forms of simulation training and not specifically VR training found a moderately favourable improvement in the time to task completion metric. In contrast, in studies where controls performed slightly faster in the final task [19-21], the authors noted that VR-trained participants demonstrated superior technique and precision, as reflected in higher procedural checklist scores and global rating scales. This raises the question of whether speed alone is a reliable proxy for surgical competency in technically demanding procedures. Furthermore, the diversity in simulator platforms and training tasks may have influenced the variation in the time metric. A potential ceiling effect may also have been reached in studies with orthopaedic residents, where time gains were smaller, suggesting diminishing returns at higher skill levels. Although reductions in task completion time reached statistical significance in several studies, the practical or clinical significance of these improvements is unclear, especially given the limited evidence of in vivo skill transfer.

The ASSET score, a validated and structured global rating scale for arthroscopic skill assessment, was used as a performance metric in four studies [31]. These studies consistently found no statistically significant difference in ASSET scores between VR-trained participants and controls when assessed in cadaveric settings. This may reflect the limited sensitivity or ceiling effect of structured assessment tools, a limitation previously noted in foundational studies on similar tools such as GRS [32] and OSATS [33]. The ASSET score improvement was seen in simulated settings, but not always in cadaveric tasks. For example, Wang et al. [23] showed that VR training significantly improved simulator-based ASSET scores, but this improvement did not translate into cadaveric task scores, suggesting a simulation-to-real-world translation gap. Several studies showed a trend towards better ASSET scores in the VR group without statistical significance. This could become significant with larger sample sizes or better-powered studies.

Time to task completion and ASSET scores were limited in their ability to reflect true surgical competence. Middleton et al. [20] highlighted that instrument path length, number of hand movements and motion efficiency provided a more granular assessment of technical skill. This perspective is supported in broader literature, which argues that motion analysis better reflects surgical competence in early trainees [34,35]. However, the challenge moving forward will be the standardisation of motion-based assessment metrics and ensuring cross-platform compatibility to allow meaningful comparison.

Previous reviews [12,14] generally demonstrated that simulation training improved arthroscopic skills among novice learners. However, these reviews did not differentiate between simulation modalities and thus did not isolate the unique contribution of virtual reality (VR) simulation. Our review extends the evidence specifically to VR simulation trainers for knee arthroscopy. Nonetheless, the evidence for true intraoperative skill transfer remains limited to one study. Our review reflects a growing consensus in the literature supporting the use of VR simulators in arthroscopic training. All studies have assessed clinical transferability to some degree by focusing on the extent to which simulator-acquired skills are applicable in cadaveric or human models. For instance, the most recent study by Tronchot et al. [27] showed that VR-trained junior orthopaedic residents performed significantly better than their peers in cadaveric arthroscopy, affirming the transferability of skills. This trend mirrors findings in other surgical specialties. For example, Seymour et al. [36] demonstrated that VR-trained surgical residents committed significantly fewer errors and completed laparoscopic procedures faster than their traditionally trained peers. These findings lend cross-specialty support to the effectiveness of VR training. However, similar to the studies we assessed, the results are not uniformly positive in other specialties [37].

In terms of a comparative perspective with other modalities of simulation training, the results are not consistent. VR training has comparative performance with other modalities. One study [19] found that VR-trained individuals outperformed those trained on benchtop simulators when assessed on cadaveric knees. Conversely, another study [25] found no significant difference between VR-trained and benchtop-trained groups. Interestingly, cadaveric training investigated in only one study [25] was shown to surpass VR training in skill acquisition, suggesting that tactile fidelity and anatomical realism may still provide unique educational benefits.

Our review has certain limitations. Firstly, there was significant methodological heterogeneity in the included studies, such as types of simulators used, duration of VR simulator use, type of training tasks performed and outcomes measured. This was reflected in previous similar reviews on simulation training in arthroscopic surgery [11,13,14]. This variability not only reflects the evolving nature of VR training but also reduces the generalisability of findings. Secondly, only a small number of studies (n=9) were eligible for the final review. Apart from one study [22], all studies had fewer than 50 participants despite being randomised controlled trials. Apart from two studies [21,26], recruited participants were medical students, junior residents or novices with minimal or no prior exposure to knee arthroscopy. While this may have enhanced internal validity, the participants may have been limited in their ability to show improvement in skills. Potential confounding factors such as variability in participants’ baseline arthroscopy exposure, differences in simulator fidelity, inconsistent training duration and variation in assessment environments may have influenced the reported outcomes. The level of detail describing control conditions varied across studies, which may influence the interpretation of comparative outcomes. Finally, all outcomes measured were surrogate technical metrics such as task completion time, ASSET scores and checklist completion scores, and not patient-related outcomes, limiting clinical applicability of findings.

It is worth mentioning the strengths of this review. The comprehensive search strategy, clearly defined research question and rigorous inclusion and exclusion criteria have produced a robust review and first meta-analysis on VR simulation training in knee arthroscopy. Apart from two studies, all other studies were level 1 evidence as RCTs. Heterogeneity was noted in the meta-analysis outcomes. The studies utilised different VR simulation trainers and yielded similar results, demonstrating versatility and effectiveness across systems. Most studies utilised validated assessment scores, such as the ASSET score and GRS, and demonstrated transferability of skills to cadaveric and intraoperative settings through objective performance improvements. VR simulation offers a comparable platform to cadaveric training while avoiding the logistical, ethical and resource-related constraints associated with cadaver use. Our review is the most up-to-date evaluation of current VR simulation platforms for knee arthroscopy and is relevant to contemporary surgical education.

Future research should aim to address gaps identified across the current and recent reviews [12,14]. Only two studies [21,27] were multicentre trials, and all but one study had a sample size less than 50. The impact on clinical outcomes remains unknown, with only one study investigating intraoperative performance. Larger multicentre randomised controlled trials need to be done to focus on intraoperative transfer of skills to validate the effectiveness of VR simulation training. Participants with different levels of experience should be recruited to understand how VR training can be optimally integrated into arthroscopic education. Future trials should also investigate the long-term impact of VR training, such as skills retention, and ideally evaluate patient-related outcomes. Cost analysis remains poorly investigated across most studies in VR simulation training. To date, no two VR simulators have been compared with each other in any study, possibly due to financial and institutional constraints. More than 20 different subjective and objective outcome measures were recorded across the included studies, indicating the need for standardised and validated outcomes to enable comparability. VR simulation training is currently recommended and endorsed by orthopaedic education bodies across the world. Several orthopaedic educational bodies (such as American College of Surgeons (ACS), Royal College of Surgeons of England (RCSEng), British Orthopaedic Association (BOA) and Joint Committee on Surgical Training (JCST)) endorse and explicitly identify VR simulation training as an effective training adjunct [38,39]. As a result, there is a need for a standardised training curriculum to reduce disparity when implementing VR simulation training. Educators need to identify the technical and psychomotor skills that may be most amenable to VR-based training and simulators that can provide them to create a meaningful training structure.

Knee arthroscopy in orthopaedic surgery is technically demanding with a steep learning curve [22]. A successful procedure demands a high level of dexterity, hand-eye coordination and spatial awareness on the part of the surgeon [23]. A survey by Keith et al. [40] found that 93% of orthopaedic residents were not very comfortable during their first arthroscopy, and 74% felt a VR simulator was important for arthroscopic training. Acquiring competence through traditional methods can be difficult due to limited OR time, work hour restrictions and concerns about patient safety [24], necessitating alternate training methods. Simulation training offers a viable solution through a risk-free environment for learning skills efficiently before transitioning to the real surgical setting. Simulation training is gradually emerging to be an integral part of surgical education across several specialties. Numerous studies have investigated and demonstrated the effectiveness of utilising simulation training in specialties such as general surgery [41], otorhinolaryngology [42] and urology [43]. This shift represents a growing recognition of the competency-based training model. VR simulation has the potential to play an increasingly important role in the structured training framework for surgical education, complementing or even replacing parts of the apprenticeship model [44].

## Conclusions

Traditional training models, often described as “see one, do one, teach one,” come with their own set of limitations. VR simulation bridges this gap by providing trainees with an immersive environment for risk-free learning. In summary, VR simulation shows promise as a training adjunct for knee arthroscopy, with improvements demonstrated primarily in surrogate measures; however, the limited evidence of in vivo transfer highlights the need for further robust evaluation. Conclusions drawn from this review should be considered preliminary and reflective of the current evidence gap, rather than definitive statements of clinical efficacy. Our review concludes that VR training improves knee arthroscopy performance and offers measurable transferability to cadaveric models, but evidence for intraoperative performance is limited. Further studies are needed on the sustainability potential of such technology, such as patient outcomes, costs, standardisation and equity in delivery. Ongoing innovation holds promise for making VR simulation an integral component of surgical education.
